# Breaking the one-dimensional paradigm: why cardiovascular research needs a multi-systems revolution

**DOI:** 10.3389/fphys.2025.1699532

**Published:** 2025-12-10

**Authors:** Sebastien Chaigne

**Affiliations:** 1 IHU Liryc, INSERM, U1045, CRCTB, University Bordeaux, Bordeaux, France; 2 CHU de Bordeaux, Cardiology, INSERM, U1045, CRCTB, Bordeaux, France

**Keywords:** systems biology, cardiovascular complexity, precision medicine, transdisciplinary collaboration, interoperability

## Introduction

The cardiovascular system is a structure of high precision, defined by a dynamic network in which molecular signaling, protein expression, cellular mechanisms, tissue architecture, regulatory networks and organ function, all converge toward a single ultimate goal: sustaining circulation and life. Because cardiovascular system dysfunction is the first cause of global mortality, it is a field of intense basic and clinical research. Unfortunately, many research approaches remain reductionist (it studies isolated variables to understand mechanisms in a highly controlled, simplified context) and linear, constrained by what I call a 1-dimensional vision. In this vision, the integrated system is fragmented into isolated components. Although this has led to progress in cardiovascular research it has also hindered transformative, integrative (in which data across scales and disciplines is combined and the system as a network of interacting components is modeled) and holistic progress. It is time for cardiovascular science and its financial support system to embrace a truly 3-dimensional perspective in which the inherent complexity of living systems is reflected.

## The limitations of linear research

Traditional cardiovascular research is effective at dissecting individual pathways, isolating single targets, and advancing reductionist approaches that focus on individual components. These approaches often involve studying isolated parts of the organ outside their natural physiological environment. For example, we have mapped the expression, distribution, and function of ion channels at a cellular level and have defined the effects of growth factors. Even after decades of research, the task remains incomplete. Similarly, we have engineered receptor-targeted drugs which provide benefit, but still frequently cause off-target effects. Despite the global burden of cardiovascular disease, the pharmaceutical industry has shown a decreasing interest in pharmacological cardiac research; with investment increasingly shifting towards more commercially rewarding therapeutic areas (Kamerikar and Seoane-Vazquez, 2023). These 1-dimensional approaches have yielded invaluable insights, yet they have also imposed artificial boundaries that constrain a more integrated understanding of cardiovascular disease. Indeed, when examining cardiac arrhythmias, our research remains overly fragmented. Electrophysiological activity is often studied separately from structural remodeling in the heart, in non-integrative contexts, preventing a full appreciation of how tissue changes shape of electrical signalling (Xia et al., 2006; Galli et al., 2021). In addition, we examine ion channel modulation pathways *in vitro*, often independently of neuro-hormonal influences, while overlooking the relative contribution of key intracellular regulators of cardiac function; moreover, genetic predisposition is frequently assessed without fully accounting for environmental influences. This is partly due to the way cardiovascular research is organised. Each research laboratory operates autonomously, investigating specialized questions within disciplines that remain overly compartmentalized (Coronel, 2020). This research provides a fragmented view that fails to capture the dynamic interplay between the heart’s electrical, structural, functional, metabolic, inflammatory, and environmental systems of the heart (multi-dimensional research). This limits our understanding of arrhythmias and hinders the development of effective therapeutic strategies.

## Two-dimensional research and its lack of integrative perspective

Two-dimensional approaches aim to integrate various aspects of cardiac function, for example, by combining electrophysiological studies with neuro-hormonal analyses. Nevertheless, these approaches remain restricted by oversimplified, compartmentalized models that neglect critical elements, including fluctuating hormone ratios, regional innervation of the heart, and local tissue microenvironments. While clinically relevant large animal models (Garry et al., 2025; Wang et al., 2025; Staelens et al., 2025; Goutchtat et al., 2025), such as swine (Jia et al., 2024), are the preferred model for testing new and novel therapeutic targets for cardiovascular disease, and are crucial for understanding disease progression, these models do not accurately reflect the interplay between human genetics, environmental, and systemic factors, including individual differences in stress, anxiety, and disease responses. Further, although more integrated interorgan investigations are possible in large animal studies (Deregowska et al., 2025), many are still performed in isolation. This lack of integration is particularly evident when translating experimental findings into clinical practice: the absence of multiscale interactions and physiological feedback complicates and often restricts the development of effective therapies. Consequently, two-dimensional research fails to capture the emergent properties of the living heart. These limitations underscore the urgent need to move beyond one- and two-dimensional paradigms toward a multi-dimensional research framework.

## Three-dimensional complexity

**FIGURE 1:**
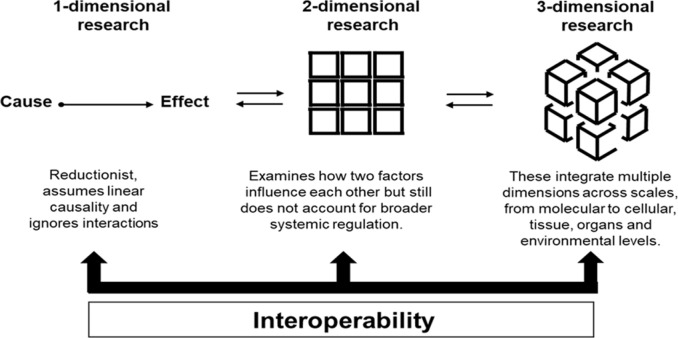
Comparative dimensional research levels.

Three-dimensional- integrative- thinking in cardiovascular research implies recognizing that biological systems operate through networks of interdependent relationships rather than through linear cause-and-effect chains (Table 1; Figure 1). The heart is not only a pump: it is a mechanosensitive organ whose electrical activity shapes metabolic pathways, whose contractile function likely modulates gene expression, and whose rhythmic cycles may regulate systemic inflammation (Darkow et al., 2023; Taghdiri, 2024), while the reverse is also true: mechanical function may modulate electrical function (Chen-Izu et al., 2025) and inflammation may modulate the cardiac cycle (Quinn and Kohl, 2021). What holds true for cardiovascular research applies equally across all areas of biomedical science. At the molecular/cellular levels, this requires awareness that proteins operate within dynamic complexes, that metabolic pathways form interconnected networks, and that cellular responses emerge from the integration of multiple signals, and that organ function is the consequence of the integration of the entirety of cellular and inter-organ cross-talk (Dong et al., 2025). Thus, at the tissue level, a 3-dimenional approach requires appreciation of how mechanical forces, electrical gradients, neuronal modulation and chemical signals generate feedback loops that either maintain physiological homeostasis or drive pathological phenotypes. Despite substantial investments over the past several years, there are numerous questions regarding cardiovascular physiology and pathophysiology which remain unanswered. In the field of cardiac mechanobiology, cardiomyocyte cells sense shear stress and translate mechanical forces into biochemical signals that regulate cardiac contraction, inflammation, and remodeling (Liu et al., 2022). While mechanobiology has broadened cardiovascular research beyond traditional biochemical frameworks, its compartmentalized approach still fails to capture the cumulative and time-dependent impact of aging effect and disease on the heart.

**TABLE 1:** Comparison of One-Dimensional versus three-dimensional approaches in cardiovascular research.

Aspect	One-dimensional approach	Integrative (3-dimensional) approach	References
Research Structure	Focus on isolated variables, reductionist, linear cause-effect	Network-based, multi-scale, holistic models; considers interactions and feedback loops	Yoshie et al. (2020), Joshi et al. (2021)
Data Interpretation	Hypothesis-driven by manual interpretation, low-dimensional	Systems level, Integrated multi-omics data, high-dimensional	Yu et al. (2023), Reitz et al. (2023)
Methodology	Use of Targeted, isolated experimental techniques	Network theory, machine learning, multi-physics modeling	Joshi et al. (2021), Sobitov et al. (2025)
Clinical Application	Standardized treatments based on isolated risk factors	Personalized and predictive medicine, novel biomarker discovery	Khan et al. (2024), DeGroat et al. (2024)

## Bringing together independent laboratory units

Pairing clinically relevant whole animal and human physiology/pathophysiology studies with multi-omics approaches offers a more promising path forward. By integrating genomics, proteomics, metabolomics, and epigenomics, we can start mapping the molecular networks that support cardiovascular function. Single-cell sequencing technologies may reveal cellular heterogeneity within cardiac tissues, which is mainly invisible to bulk analyses. Spatial transcriptomics show how gene expression patterns generate functional gradients throughout the heart. Computational modeling and artificial intelligence (AI), though still underutilized, will provide essential tools to integrate experimental data across scales, from molecular interactions to cellular networks, tissue architecture, and organ-level function, thereby offering the cohesive insight needed to understand cardiac physiology. To realize their full potential, it is crucial to ensure the interoperability of datasets, analytical platforms, and models, so that information can flow and combine without any loss of value. Digital twins of patients, combining mathematical modeling, AI, explainable AI and interoperable data, could 1 day guide personalized therapies by predicting the effect of interventions on complex disease networks. Bridging these dimensions requires unifying previously independent research labs into a hub or laboratories, fostering not only interdisciplinary but also extradisciplinary approaches in which research methods from other research fields are applied (Li et al., 2025).

## Clinical translation through integrative 3-dimensional cardiovascular systems approaches

Three-dimensional, integrative approaches are already demonstrating their potential across multiple domains (Amar et al., 2018; Thomas and Wu, 2025). For example, precision medicine initiatives employ multi-parameter risk prediction models that integrate genetic, environmental, and physiological data to identify patients at risk of cardiovascular events. These approaches should outperform traditional risk factors because they capture the multidimensional nature of disease susceptibility. Network-based drug discovery identifies combination therapies that target multiple nodes/targets within disease networks rather than single pathways (Tan et al., 2022). Early results suggest that these strategies can overcome the limitations of single-target therapeutics (Ryszkiewicz et al., 2025). It is now recognized that cardiac repair requires the coordinated orchestration of multiple processes including stem cell differentiation, angiogenesis, immune modulation, and mechanical support. Success depends on harmonizing these systems rather than optimizing individual isolated components (Manoria, 2025). Taken together, these advances underscore the need to re-evaluate cardiovascular research through an integrative, system-wide lens that bridges molecular mechanisms with clinical outcomes.

## A Plea for Transformation of Research and its funding

The transition from 1-, 2- to 3-dimensional cardiovascular research will require profound institutional change. Future research laboratories should merge traditional approaches with cutting-edge technologies- from bio-printing and tissue engineering to digital twin platforms - bring together diverse expertise,collaborate with industry, and remain flexible for long-term, high-risk, high-reward projects. The goal is to generate dynamic, predictive models of the heart that encompass molecular flux, organ-level function, inter-organ cross talk and real-time physiological feedback. Looking ahead, research should move beyond 3-toward 4-dimensional research, integrating developmental trajectories and disease progression to capture the full temporal complexity of cardiovascular systems. Universities should establish extradisciplinary 3-dimensional research structures that transcend traditional departmental boundaries within the same centre, rather than reinforcing fragmented laboratories. Such structures would allow researchers to devote 100% of their time to fundamental discovery for the benefit of patients rather than chasing funding. Scientific journals should prioritize studies that integrate multiple levels of biological organization. Funding agencies will preferably support interdisciplinary 3-dimensional collaborations and long-term 3-dimensional systems projects, with evaluation frameworks suited to the complexity and scope of such initiatives. Equally essential is training a new generation of cardiovascular scientists and clinicians comfortable with emerging technologies, capable of integrating results from multiple disciplines and confident in pursuing high-risk, high-reward research without career penalties. Along the same lines, graduate programs should embrace more multi-dimensional, translational, integrative training programs, moving beyond narrowly unidirectional programs dependent upon a single field of expertise.

## Conclusion

Therefore, cardiovascular research must evolve beyond reductionist paradigms by integrating molecular, cellular, tissue and systemic perspectives from both clinically relevant animal and human studies. Technological advances in imaging and computational modelling, as well as single-cell multi-omics, provide unprecedented opportunities to capture the complexity of health and disease. Furthermore, patient-specific approaches that combine precision medicine with systems biology may help to tailor interventions more effectively. These strategies should deepen our mechanistic understanding and bridge the gap between bench and bedside. Addressing cardiovascular disease as a multidimensional challenge will ultimately enable the development of innovative, clinically transformative therapies.
